# Supplementary material for: Integrative Multi-OMICs Identifies Therapeutic Response Biomarkers and Confirms Fidelity of Clinically Annotated, Serially Passaged Patient-Derived Xenografts Established from Primary and Metastatic Pediatric and AYA Solid Tumors
Source: Cancers (Basel). 2022 Dec 30;15(1):259. doi: 10.3390/cancers15010259 (PMC9818438; doi:10.3390/cancers15010259)
Supplement: Supplementary file 1 [file cancers-15-00259-s001.zip › Supplementary Figs and Tables/Suppplementary Figs only.pptx]

## Slide 1
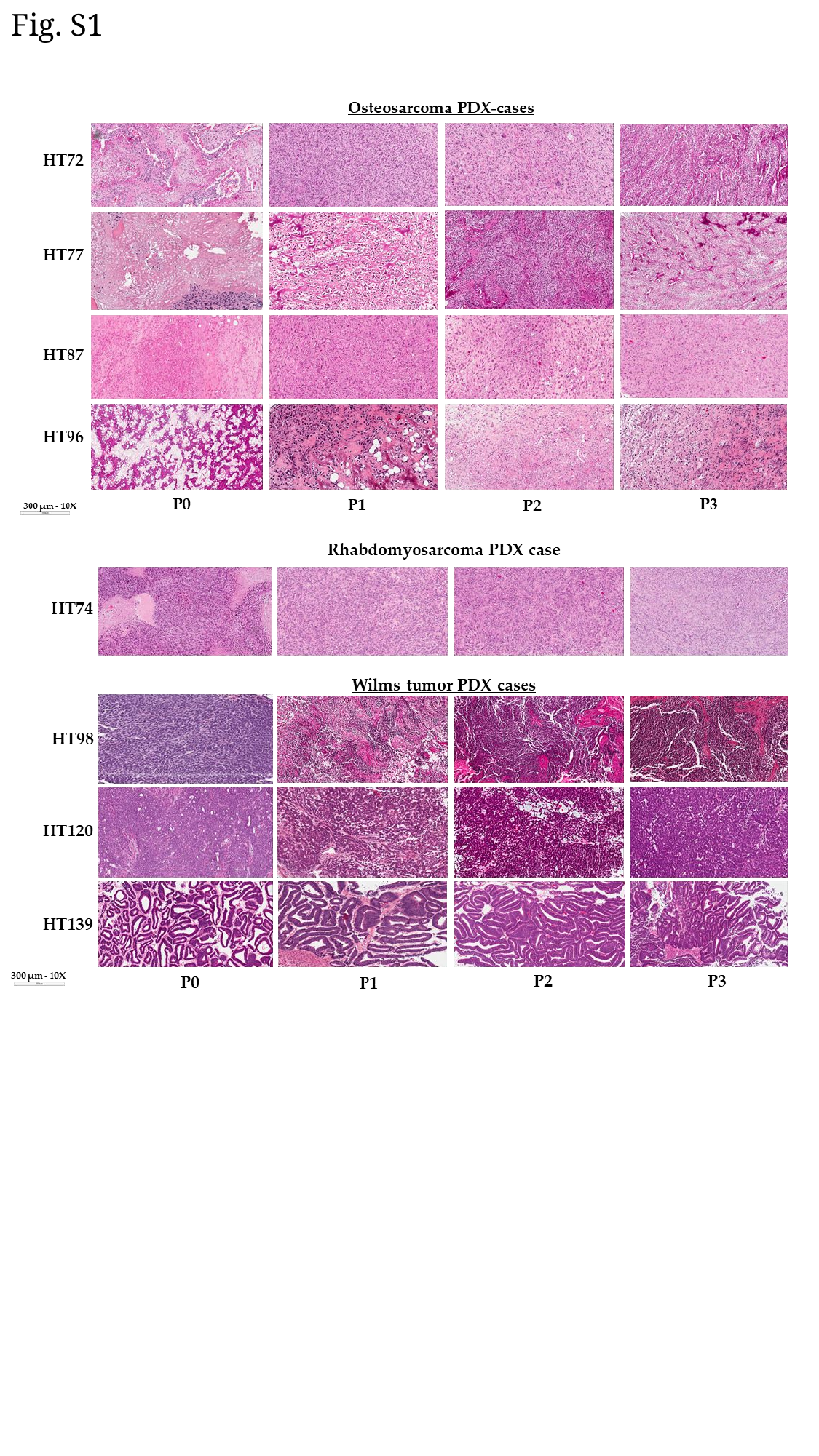

Fig. S1

## Slide 2
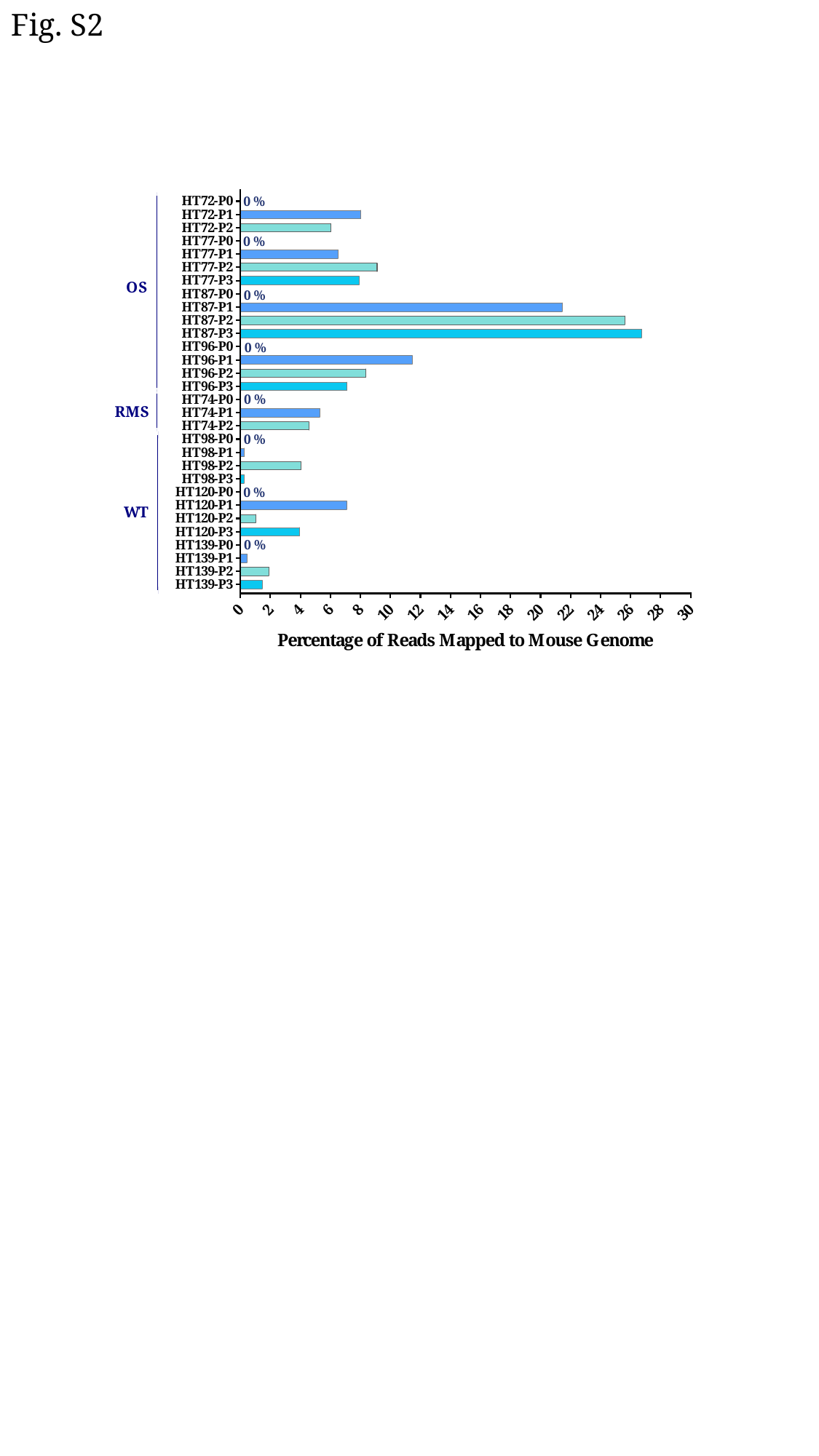

Fig. S2

## Slide 3
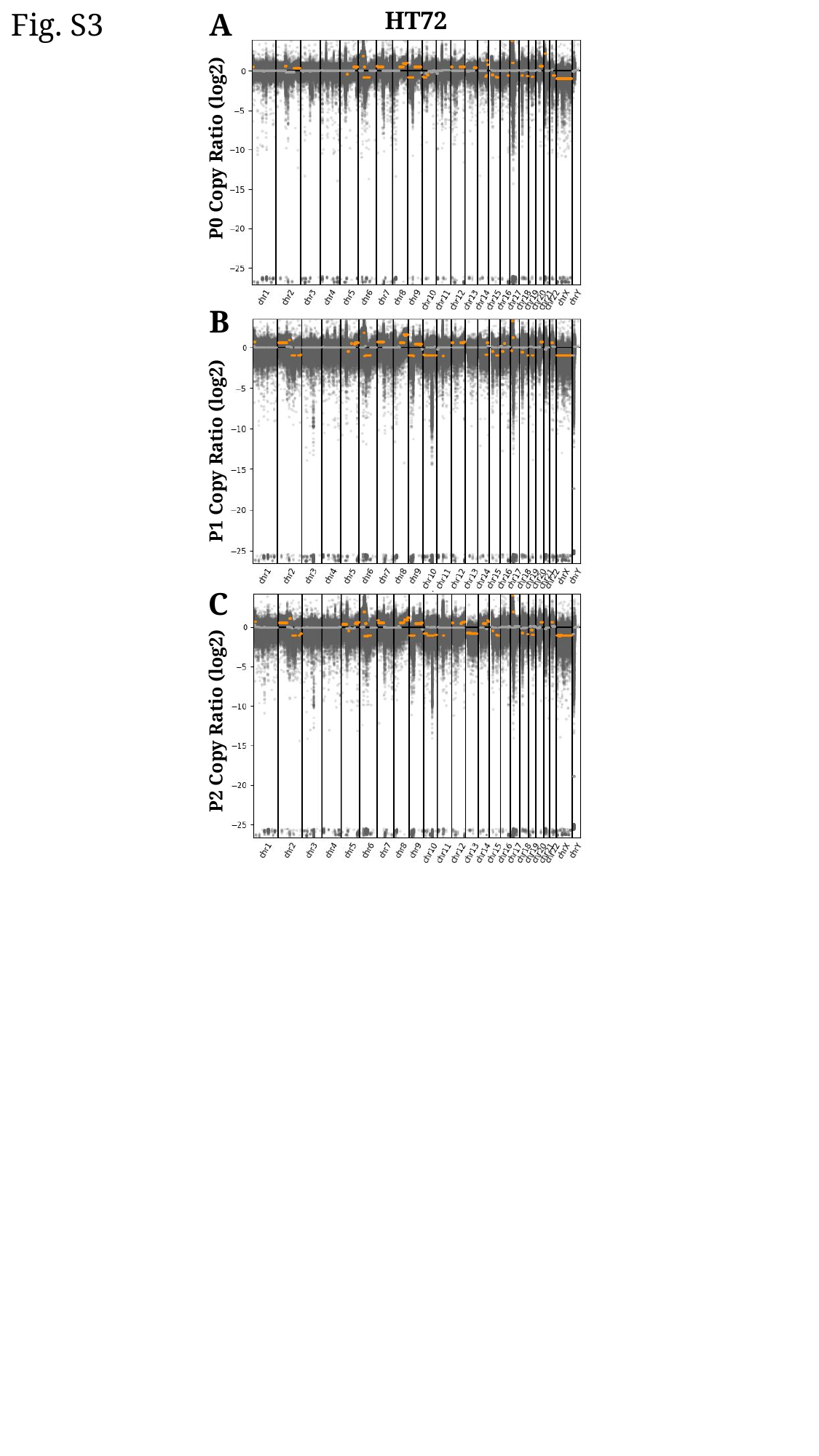

Fig. S3
A
HT72
P0 Copy Ratio (log2)
B
P1 Copy Ratio (log2)
C
P2 Copy Ratio (log2)

## Slide 4
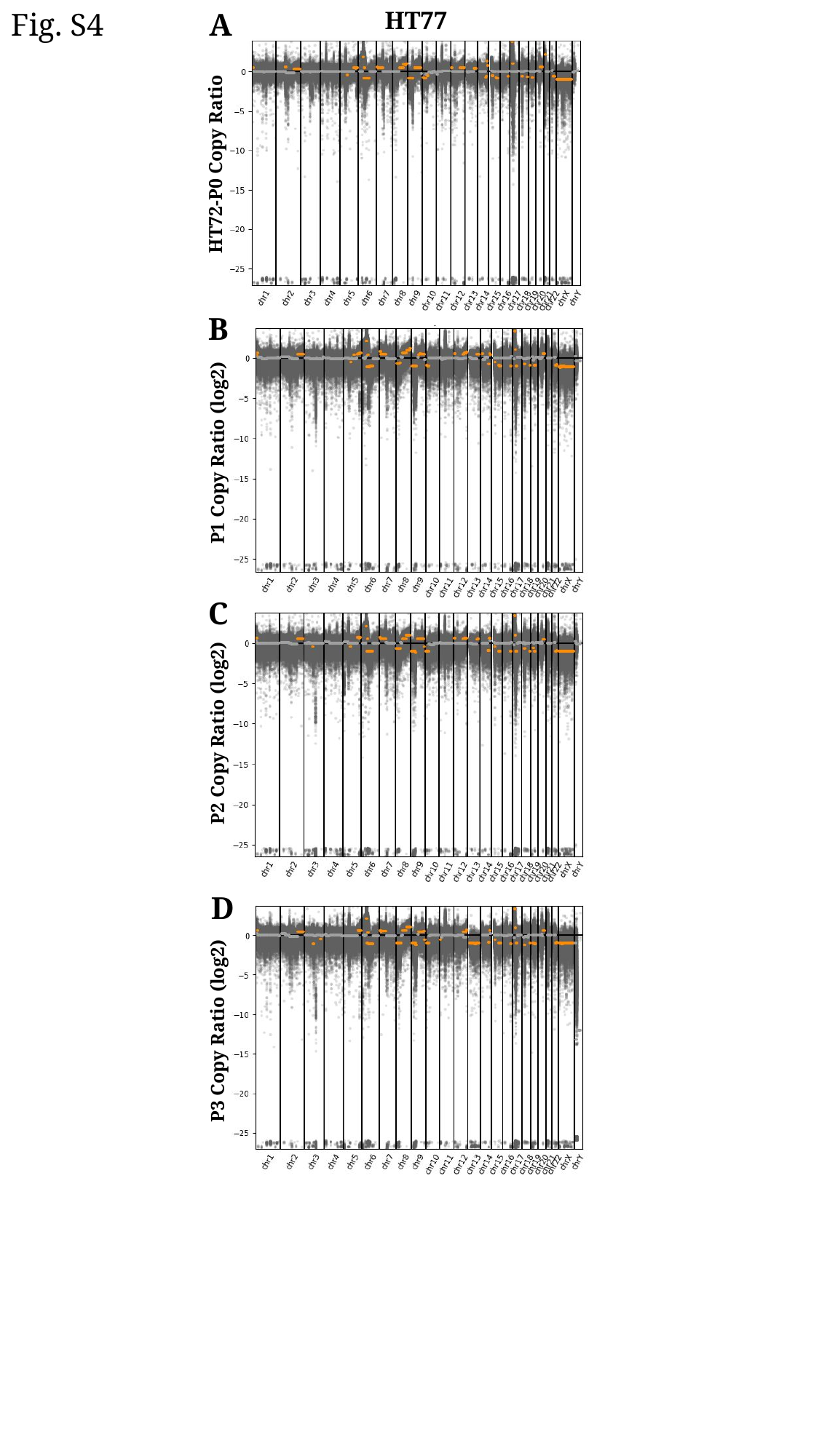

Fig. S4
A
HT77
HT72-P0 Copy Ratio (log2)
B
P1 Copy Ratio (log2)
C
P2 Copy Ratio (log2)
D
P3 Copy Ratio (log2)

## Slide 5
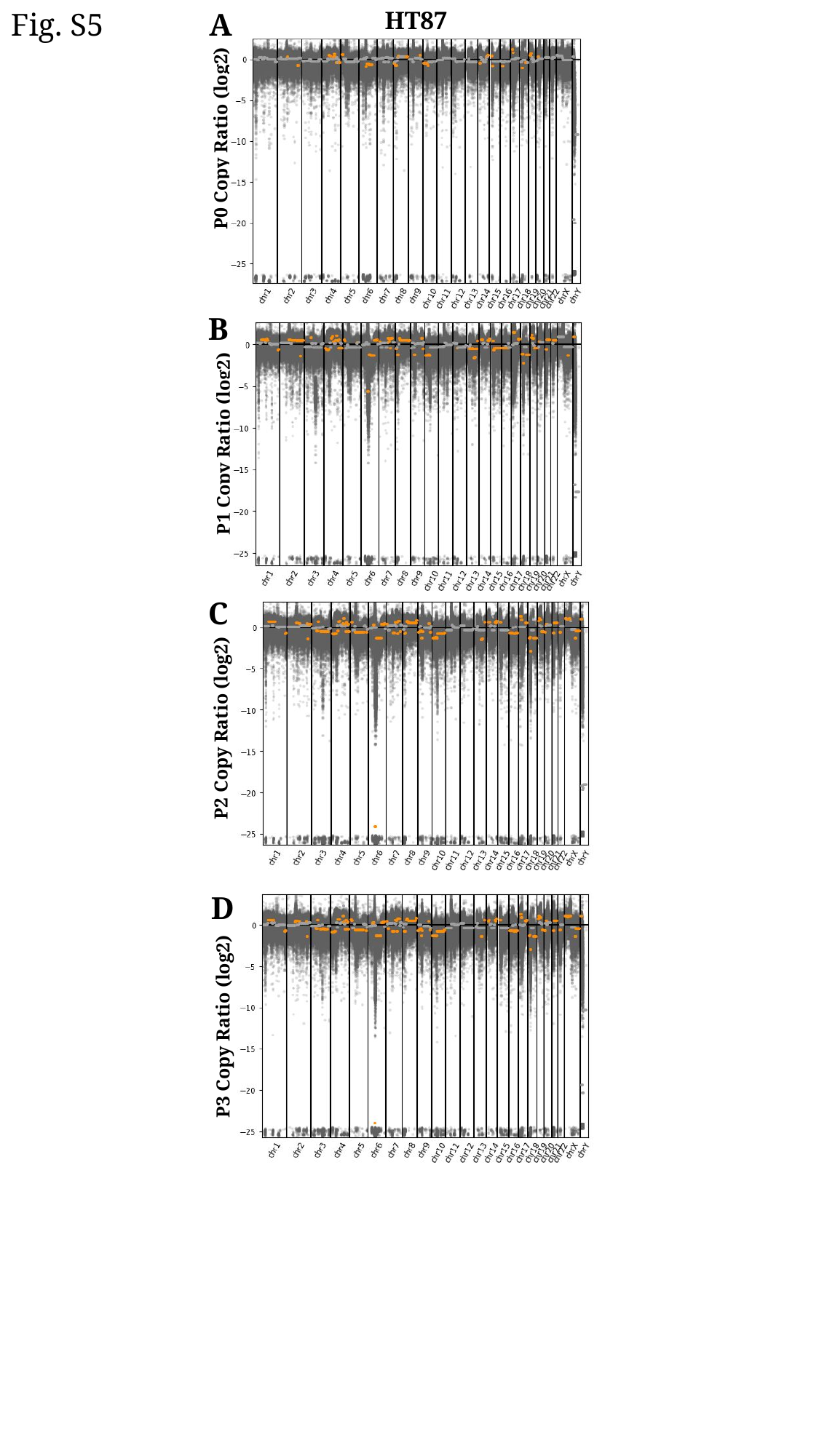

Fig. S5
A
HT87
P0 Copy Ratio (log2)
B
P1 Copy Ratio (log2)
C
P2 Copy Ratio (log2)
D
P3 Copy Ratio (log2)

## Slide 6
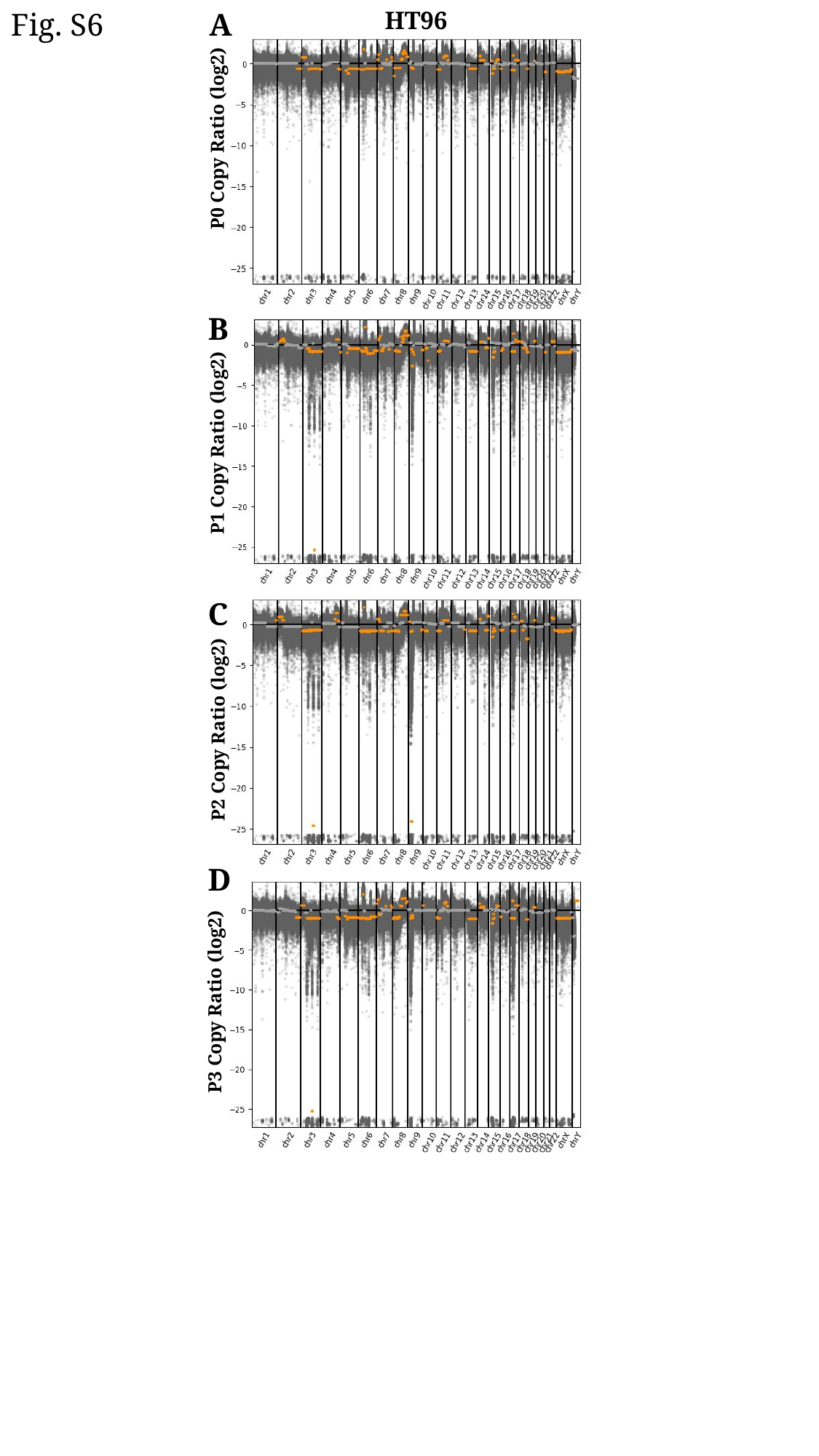

Fig. S6
A
HT96
P0 Copy Ratio (log2)
B
P1 Copy Ratio (log2)
C
P2 Copy Ratio (log2)
D
P3 Copy Ratio (log2)

## Slide 7
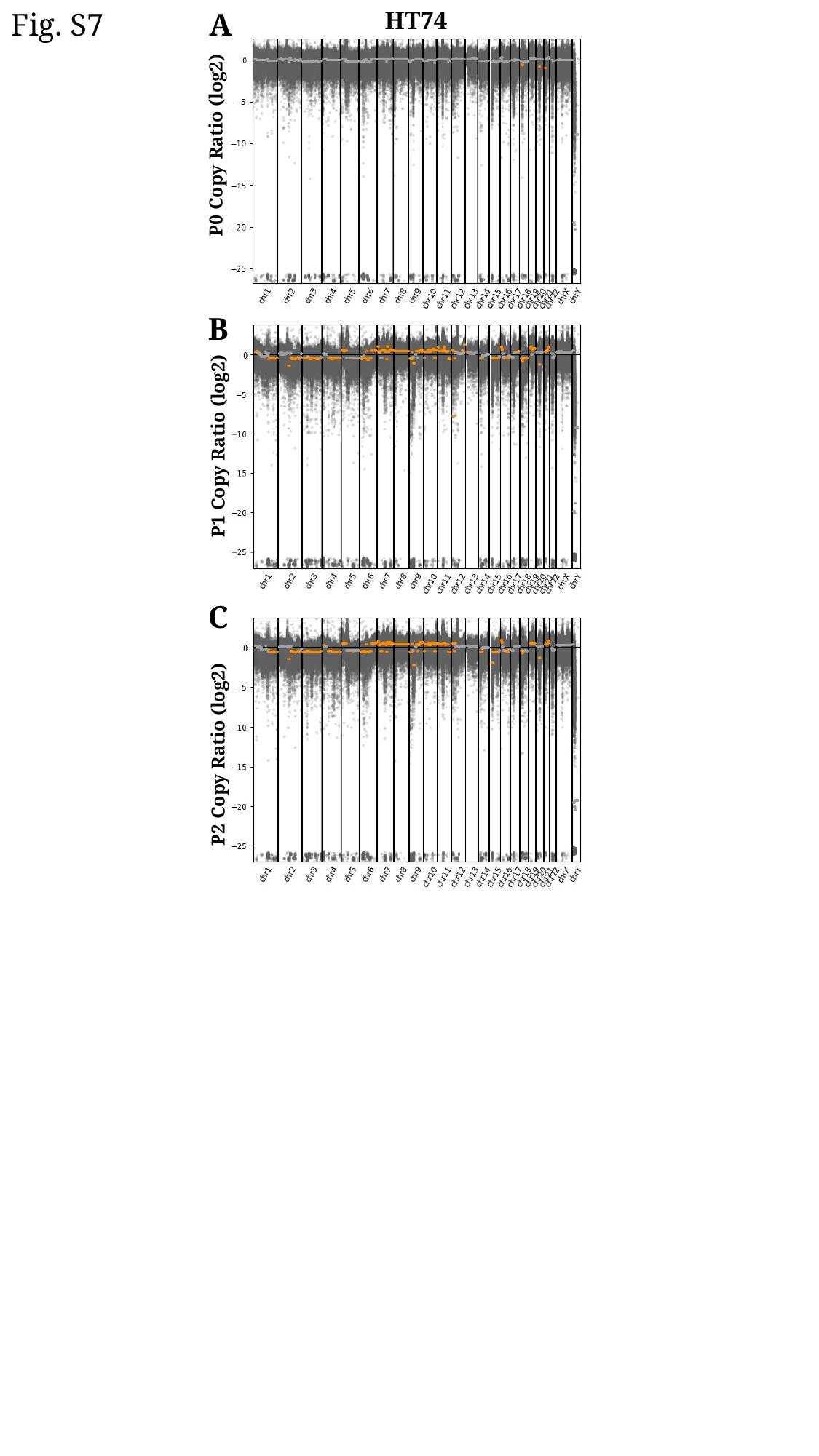

Fig. S7
A
HT74
P0 Copy Ratio (log2)
B
P1 Copy Ratio (log2)
C
P2 Copy Ratio (log2)

## Slide 8
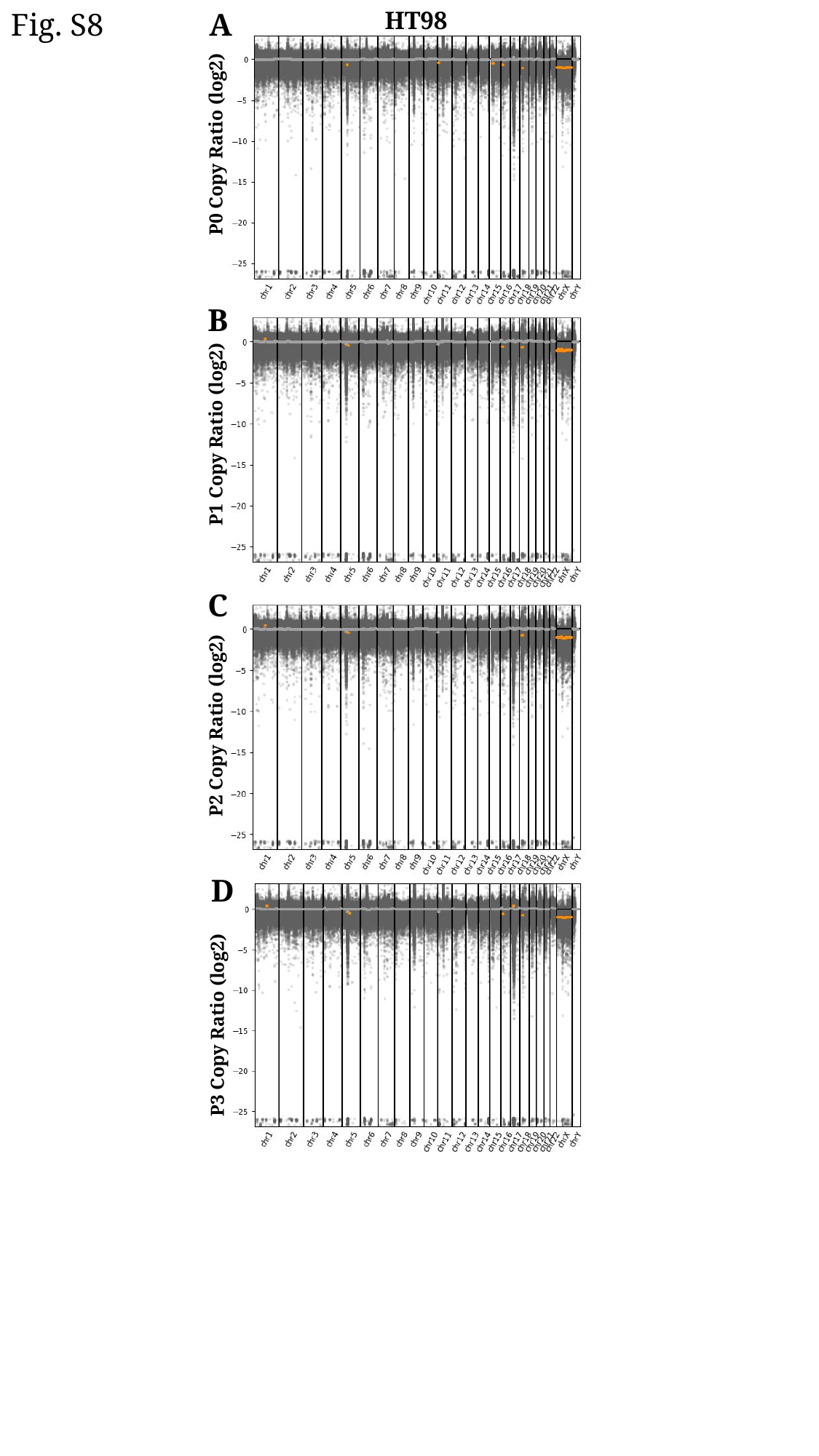

Fig. S8
A
HT98
P0 Copy Ratio (log2)
B
P1 Copy Ratio (log2)
C
P2 Copy Ratio (log2)
D
P3 Copy Ratio (log2)

## Slide 9
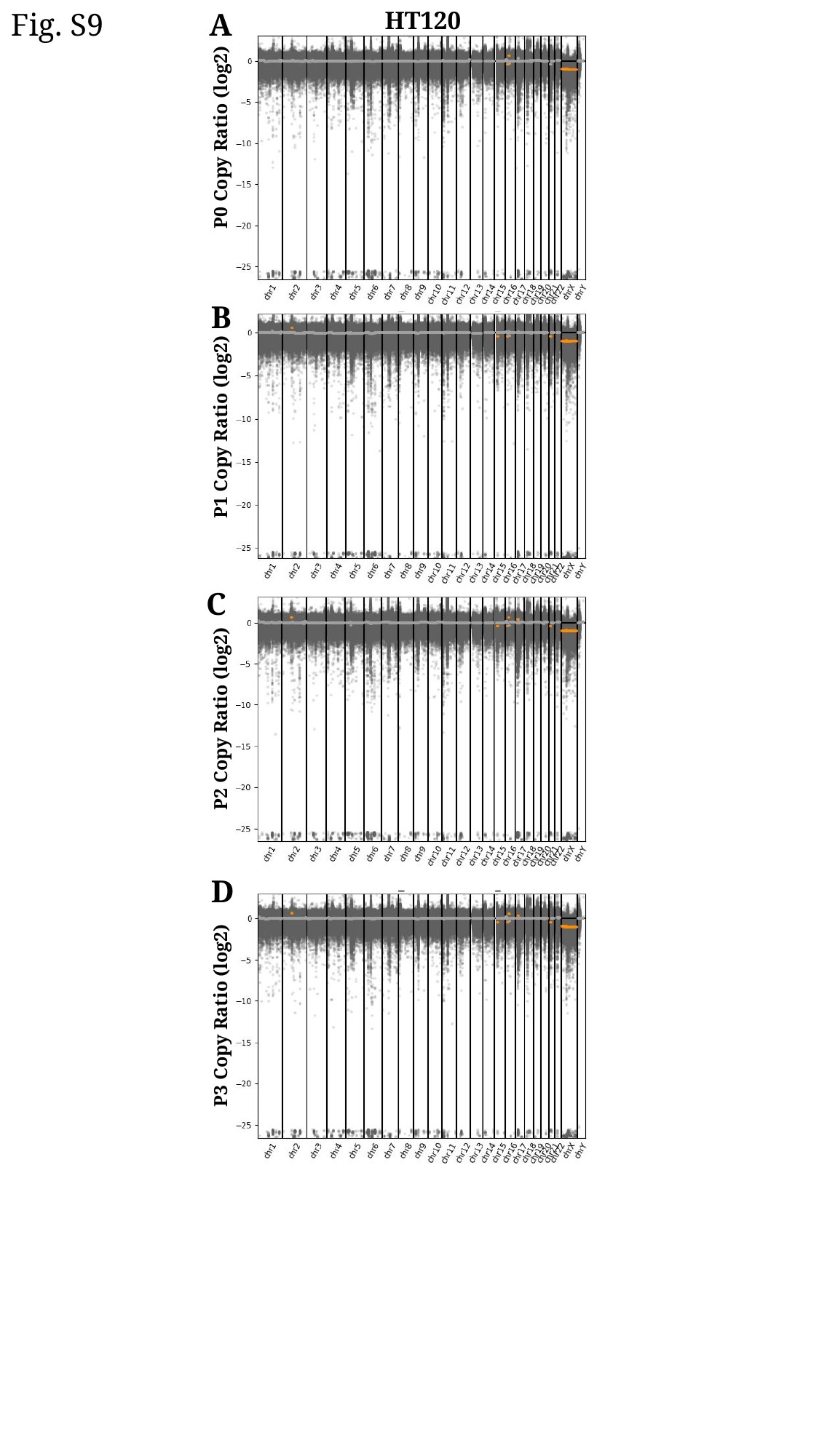

Fig. S9
A
HT120
P0 Copy Ratio (log2)
B
P1 Copy Ratio (log2)
C
P2 Copy Ratio (log2)
D
P3 Copy Ratio (log2)

## Slide 10
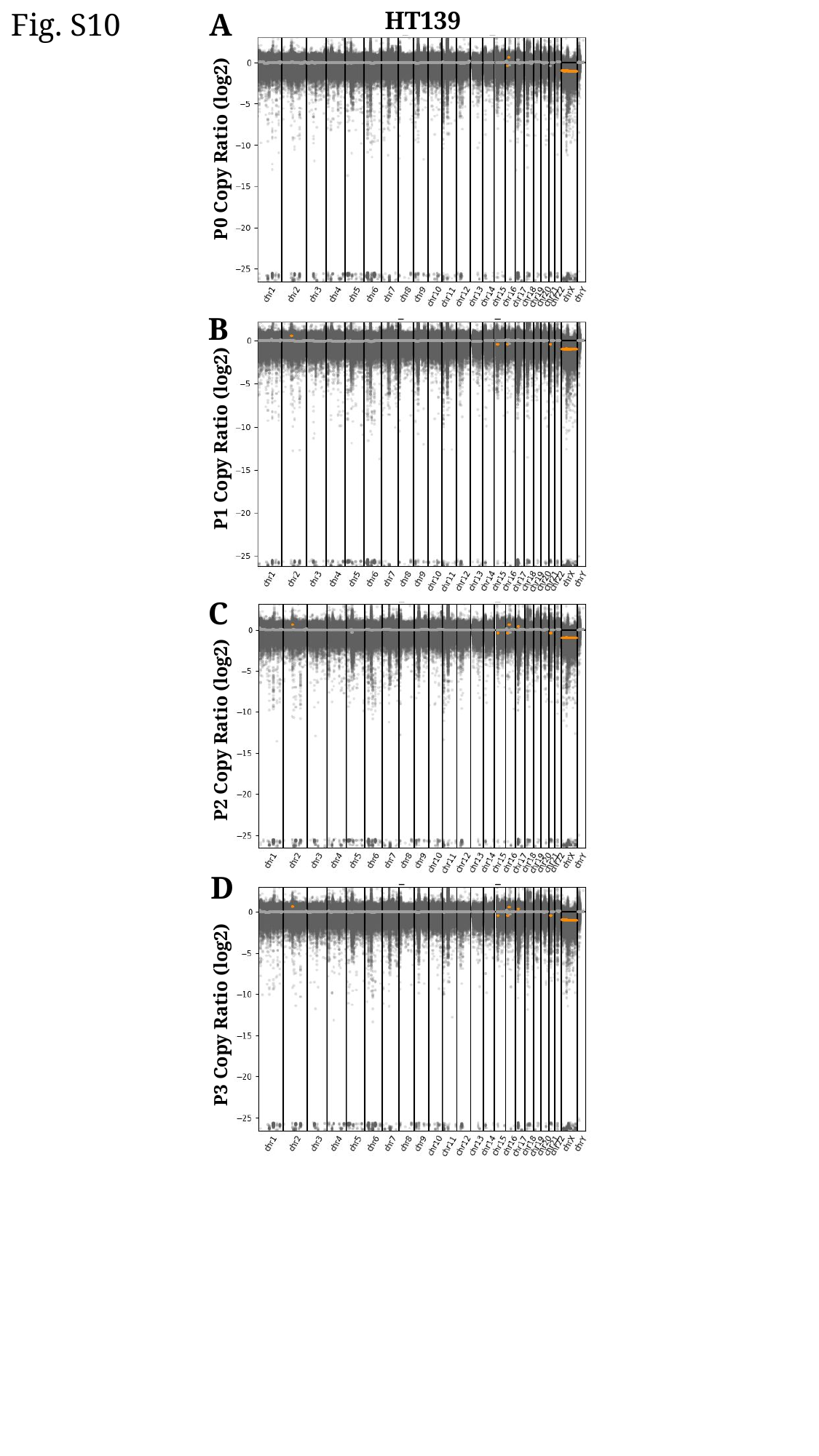

Fig. S10
A
HT139
P0 Copy Ratio (log2)
B
P1 Copy Ratio (log2)
C
P2 Copy Ratio (log2)
D
P3 Copy Ratio (log2)

## Slide 11
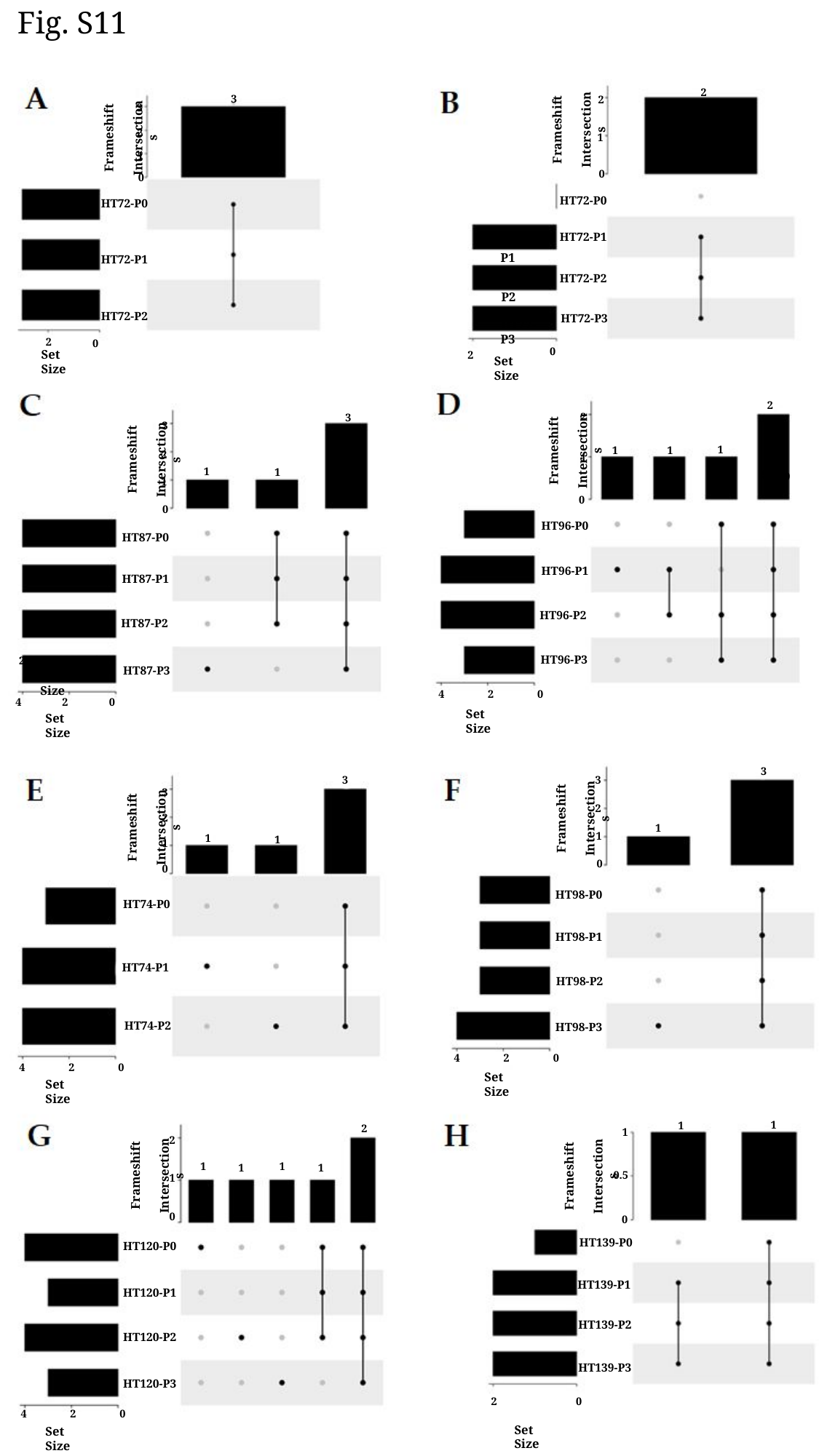

Fig. S11
2
3
Frameshift
 Intersections
1
0
21
41
HT72-P0
HT77-P1
HT72-P1
HT77-P2
HT72-P2
HT77-P3
2
0
Set Size
Set Size
2
1
0
3
1
93
29
0
HT96-P0
HT87-P0
HT96-P1
HT87-P1
HT96-P2
HT87-P2
HT96-P3
0
0
2000
1000
1000
HT87-P3
Set Size
Set Size
3
8
1
1
HT98-P0
HT74-P0
HT98-P1
HT74-P1
HT98-P2
HT74-P2
HT98-P3
Set Size
HT139-P0
HT120-P0
HT139-P1
HT120-P1
HT139-P2
HT120-P2
HT139-P3
HT120-P3
2
3
Frameshift
 Intersections
2
1
0
HT72-P0
HT72-P1
HT72-P2
HT72-P3
0
2
2
3
Frameshift
 Intersections
1
1
1
Frameshift
 Intersections
2
1
1
4 2 0
4
2
0
Set Size
3
3
3
2
1
0
2
Frameshift
 Intersections
Frameshift
 Intersections
1
1
0
4 2 0
4 2 0
Set Size
1
1
2
1
2
1
0
1
1
Frameshift
 Intersections
1
1
Frameshift
 Intersections
0.5
0
2 0
4 2 0
Set Size
Set Size

## Slide 12
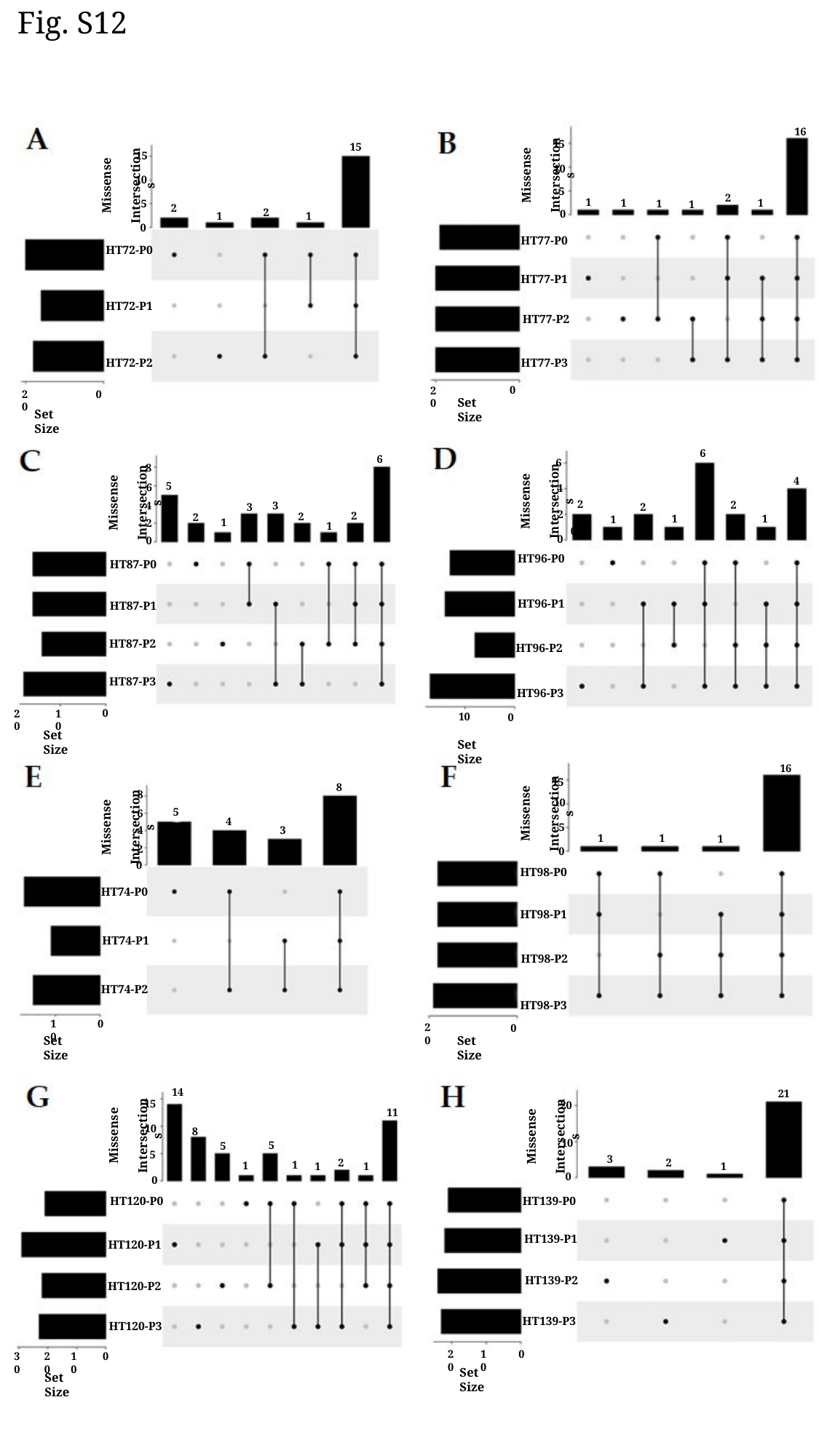

Fig. S12
16
15
15
15
Missense
 Intersections
0
2
2
2
HT72-P0
HT72-P1
HT72-P2
20
0
Set Size
Set Size
6
4
5
2
0
1
0
HT96-P0
HT87-P0
HT96-P1
HT87-P1
HT87-P2
HT96-P2
HT87-P3
0
1000
1000
HT96-P3
0
Set Size
Set Size
3
8
HT98-P0
HT74-P0
HT98-P1
HT74-P1
HT98-P2
HT74-P2
HT98-P3
HT120-P0
HT139-P0
HT139-P1
HT120-P1
HT139-P2
HT120-P2
HT139-P3
HT120-P3
Set Size
Missense
 Intersections
10
10
5
1
1
1
1
5
1
0
1
1
HT77-P0
HT77-P1
HT77-P2
HT77-P3
0
20
6
6
8
6
4
Missense
 Intersections
Missense
 Intersections
2
4
3
2
3
2
2
2
2
1
1
1
2
1
0
0
10
20
10
16
15
8
8
10
Missense
 Intersections
5
6
Missense
 Intersections
4
5
3
4
1
1
1
2
0
0
10
0
20
0
Set Size
Set Size
14
21
15
20
11
Missense
 Intersections
10
Missense
 Intersections
8
10
5
5
5
3
2
2
1
1
1
1
1
0
0
20
10
0
30
20
10
0
Set Size

## Slide 13
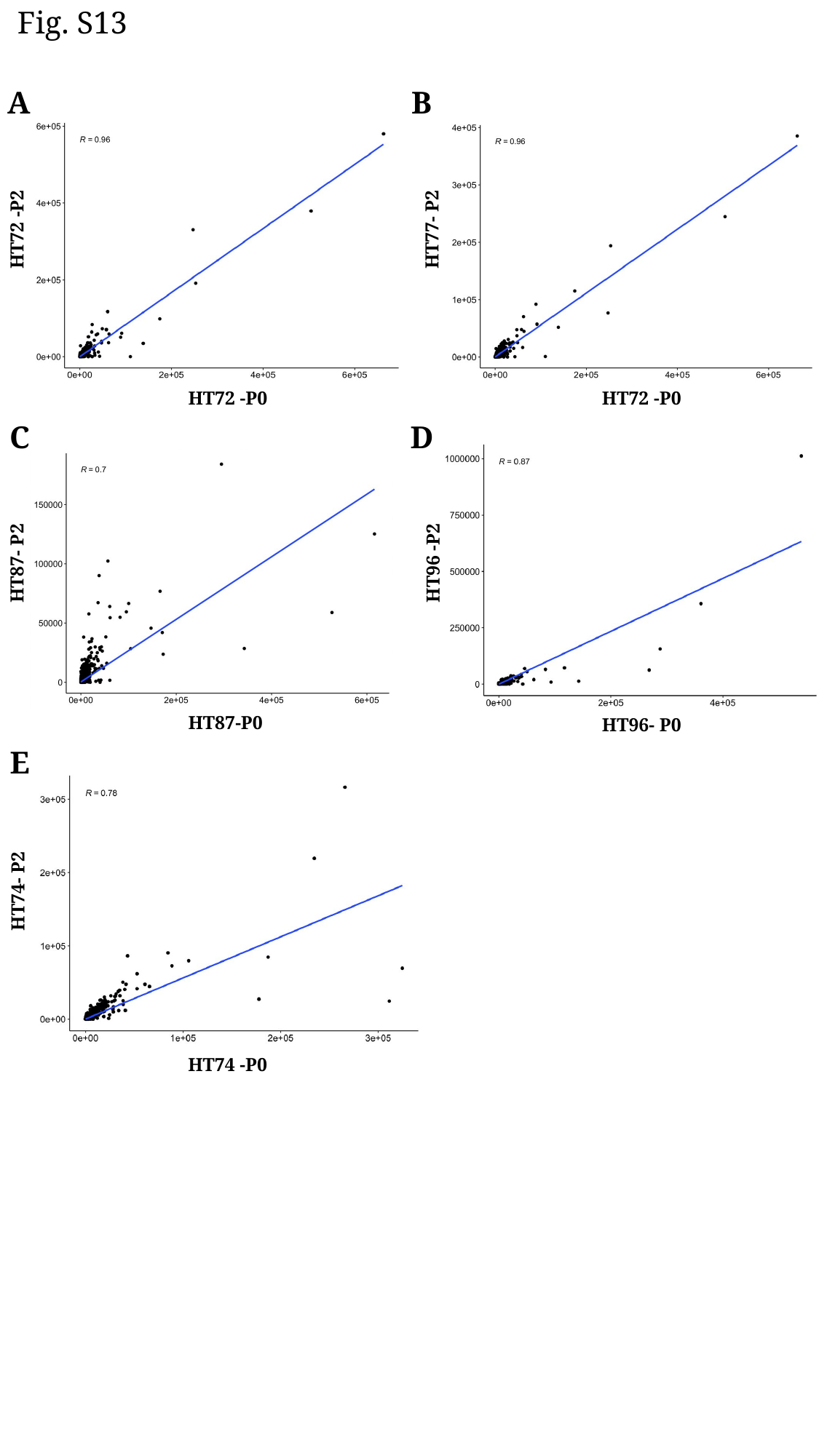

Fig. S13
A
B
HT77- P2
HT72 -P2
HT72 -P0
HT72 -P0
C
D
HT96 -P2
HT87- P2
HT87-P0
HT96- P0
E
HT74- P2
HT74 -P0

## Slide 14
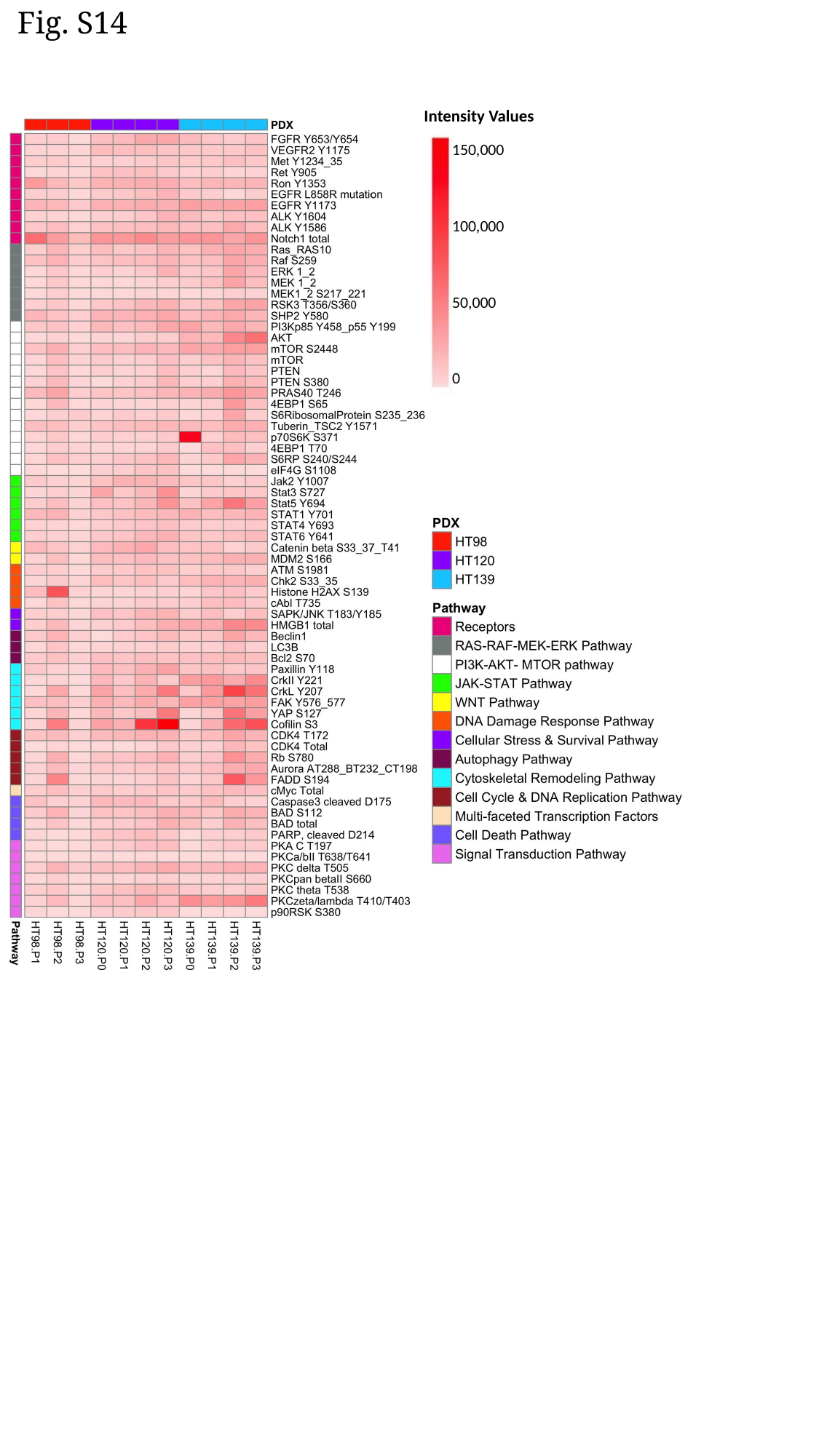

Fig. S14
Intensity Values
150,000
100,000
50,000
0

## Slide 15
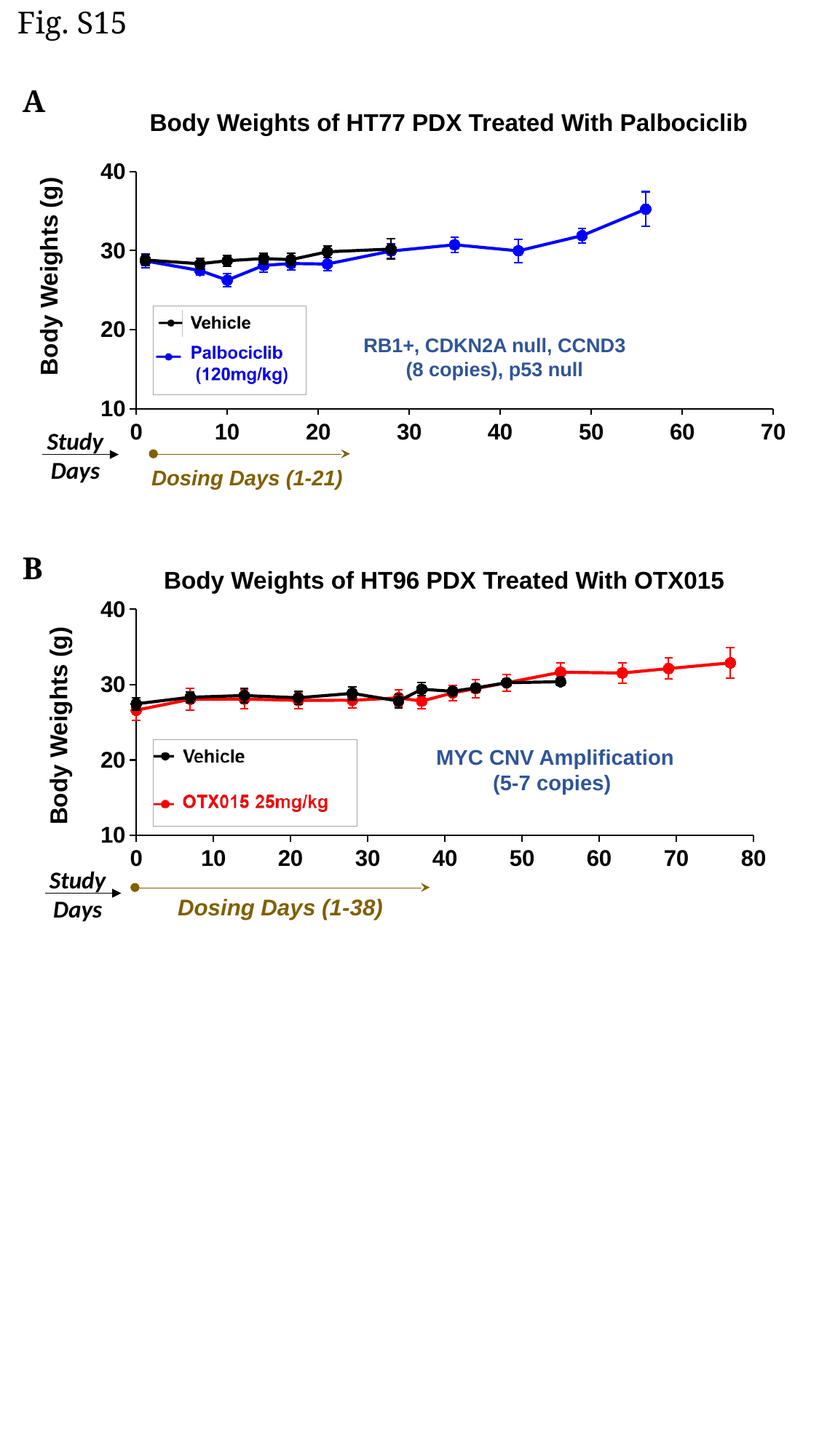

Fig. S15
A
### Chart
| Category | Grp 1 Vehicle | Grp 3 Palbocicilib 120mg/kg |
|---|---|---|Body Weights of HT77 PDX Treated With Palbociclib
Body Weights (g)
RB1+, CDKN2A null, CCND3 (8 copies), p53 null
Study Days
Dosing Days (1-21)
B
Body Weights of HT96 PDX Treated With OTX015
### Chart
| Category | Group 1 - Vehicle | Group 3- 25 mg/kg OTX-015 |
|---|---|---|Body Weights (g)
MYC CNV Amplification
(5-7 copies)
Study Days
Dosing Days (1-38)
